# Dulaglutide-associated interstitial granulomatous dermatitis: A case report

**DOI:** 10.1016/j.jdcr.2026.09.009

**Published:** 2026-09-15

**Authors:** Georgio Chidiac, Carole Kesrouani, Joelle Korkomaz

**Affiliations:** a School of Medicine and Medical Sciences, Holy Spirit University of Kaslik, Jounieh, Lebanon; b Department of Dermatology, Notre Dame des Secours University Hospital, Byblos, Lebanon; c Department of Dermatology, Saint George Hospital University Medical Center, Saint George University of Beirut, Beirut, Lebanon; d Department of Dermatology, Keserwan Medical Center - American University of Beirut Medical Center, Ghazir, Lebanon; e Department of Pathology, Hotel Dieu de France Hospital, Beirut, Lebanon

**Keywords:** dulaglutide, glucagon-like peptide-1 (GLP-1) receptor agonist, interstitial granulomatous dermatitis (IGD), reactive granulomatous dermatitis (RGD), type 2 diabetes

## Introduction

Reactive granulomatous dermatitis (RGD) is a term proposed to describe several overlapping entities that have variable clinical and histologic features.^1^ Interstitial granulomatous dermatitis (IGD) is considered part of this spectrum and was first described in 1993 by Ackerman et al as asymptomatic palpable linear truncal cords in patients with inflammatory arthritis.^2^ This reaction has been reported in different drug classes, in particular angiotensin-converting enzyme inhibitors, tumor necrosis factor inhibitors, tocilizumab, vedolizumab and talimogene laherparepvec.^3^, ^4^, ^5^ Herein, we report a case of dulaglutide-associated IGD.

## Case

A 75-year-old male presented with a persistent asymptomatic truncal eruption for 2 weeks. His medical history revealed that he had diabetes mellitus, hypertension, dyslipidemia, and atrial fibrillation for several years. His current treatment comprised rosuvastatin, fenofibrate, rivaroxaban, telmisartan, amlodipine, eplerenone, bisoprolol, metformin, insulin and dulaglutide. Of note, dulaglutide was initiated for the management of diabetes 1 month prior to lesions onset, with no changes in his other medications. He had no history of autoimmune disease or arthritis. A review of systems was unremarkable. Skin examination showed bilateral 6 × 9 cm, non-scaly, slightly annular erythematous patches on his flanks, with well-defined margins and no induration (Fig 1), not involving dulaglutide injection sites. Hair, nail, and mucous membrane examination was normal. There was no regional lymphadenopathy. Histopathology of a skin biopsy specimen with hematoxylin and eosin (H&E) revealed interstitial and perivascular infiltrates of lymphocytes and histiocytes, scattered throughout the dermis, and forming small interstitial granulomas (Fig 2). Mucin deposition, lymphoid atypia and interface dermatitis were absent. The clinical and histologic findings were consistent with IGD. Routine blood tests were unremarkable, with no biological evidence of infectious, autoimmune, or hematological disease. Withdrawal of dulaglutide led to complete resolution of the lesions within 10 days, with no recurrence observed during a 2-year follow-up period.

**Fig 1:**
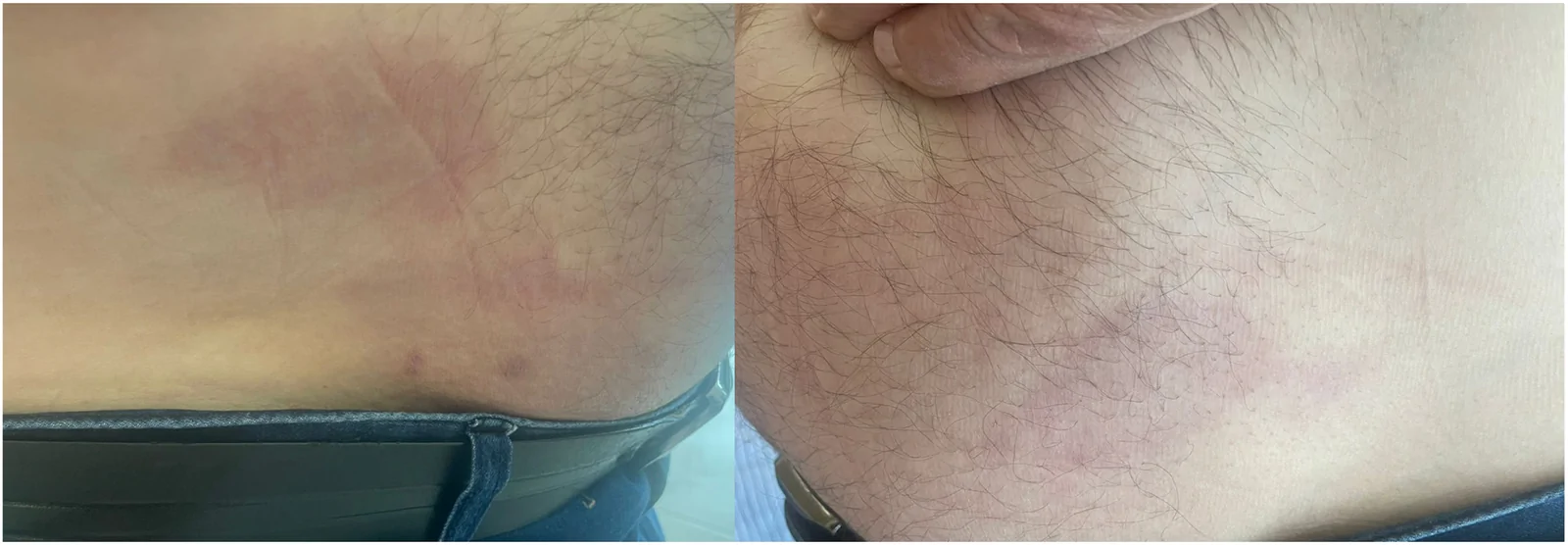
Bilateral erythematous patches measuring 6 × 9 cm, non-scaly, slightly annular, with well-defined margins on the flanks.

**Fig 2:**
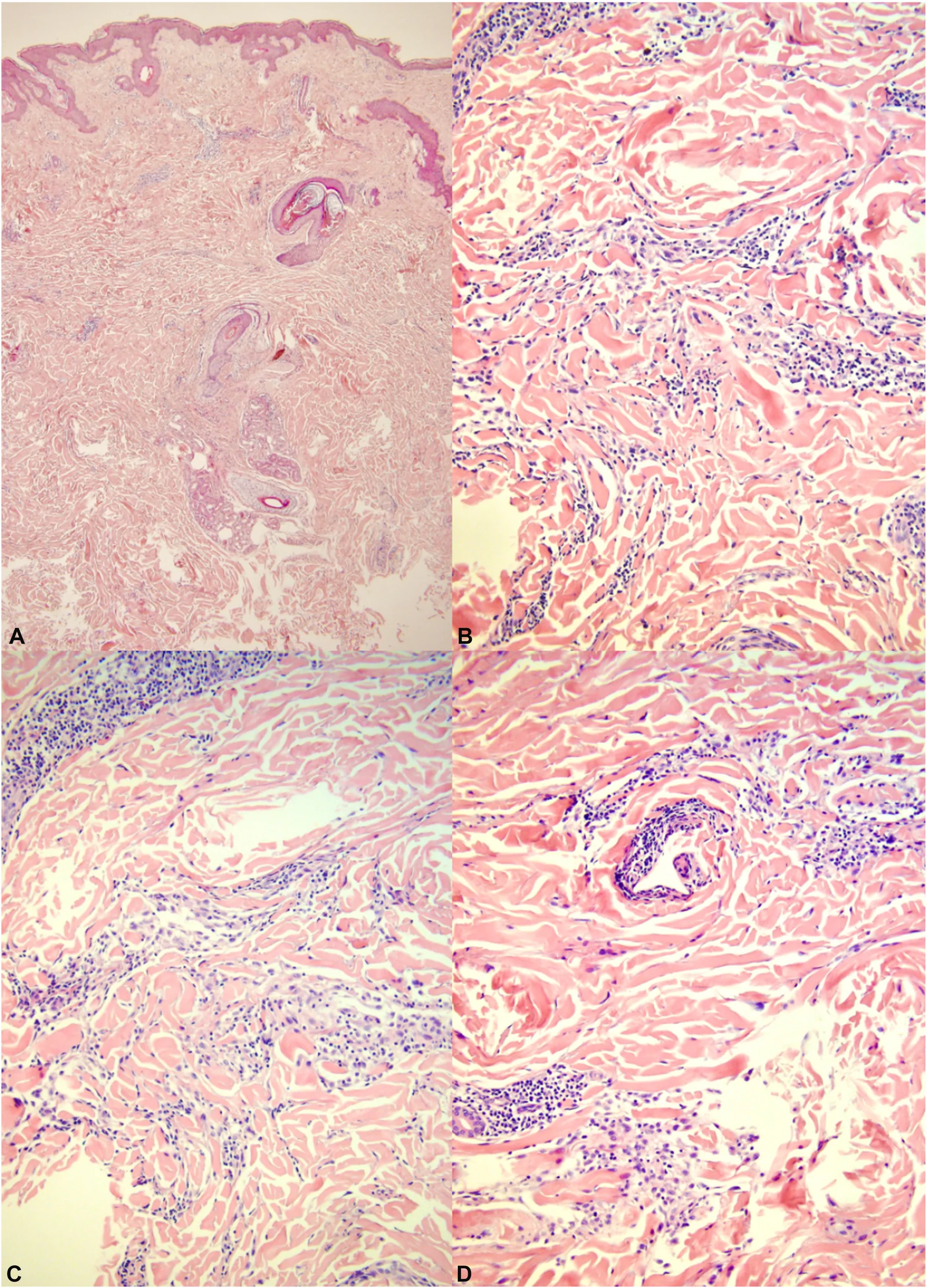
Interstitial and perivascular infiltrates of lymphocytes and histiocytes scattered throughout the dermis, forming small interstitial granulomas. **A,** Low-power magnification (hematoxylin and eosin [H&E], 4×). **B-D,** Higher-power magnification (H&E, 20×).

## Discussion

Dulaglutide, a glucagon-like peptide-1 (GLP-1) receptor agonist, is a key glucose-lowering agent for patients with type 2 diabetes. In addition to its convenient once-weekly injection regimen, it has demonstrated beneficial effects on body weight and major adverse cardiovascular events. Most common adverse effects are primarily gastrointestinal symptoms, such as nausea, vomiting, and diarrhea, and are typically mild and self-limiting. Reported dermatologic events are rare and have largely been limited to mild, localized injection-site reactions, with only a few documented cases of more generalized morbilliform drug eruptions or severe hypersensitivity reactions.^6^^,^^7^

Here, we describe a case of dulaglutide-associated IGD. IGD is a subtype of the broader category of RGD, which also includes palisaded neutrophilic and granulomatous dermatitis (PNGD) and interstitial granulomatous drug reaction (IGDR).^8^^,^^9^ Although the initial description of IGD highlighted the “rope sign” (a linear subcutaneous cord) in patients with inflammatory arthritis, more recent reports underline that IGD can present as erythematous to violaceous patches, plaques, papules, or nodules distributed on the trunk and proximal limbs. The histopathology typically reveals a heavy histiocytic infiltrate without significant mucin deposition or leukocytoclastic vasculitis, in contrast to PNGD, which often features more pronounced neutrophilic involvement and palisading granulomas, and IGDR, which characteristically shows interface changes, prominent eosinophils, and sometimes lymphoid atypia. However, these entities overlap significantly in their clinical presentations, their histological features, and their underlying conditions and are considered a continuum by many authors.^1^

RGD subtypes, including IGD, have been linked to various autoimmune conditions (eg, rheumatoid arthritis, systemic lupus erythematosus, Sjögren syndrome), as well as hematological and solid organ malignancies, and chronic infections.^10^ A subset of RGD cases are drug-induced and various cardiovascular and immunomodulatory drugs have been implicated.

In this patient, the temporal relationship, improvement following treatment discontinuation, and absence of an identifiable autoimmune or infectious trigger support a probable association between dulaglutide and the development of IGD. The Naranjo Adverse Drug Reaction Probability Scale yielded a score of 6, consistent with a probable adverse drug reaction. However, a definitive causal relationship cannot be established, particularly in the absence of drug rechallenge. This case broadens the list of agents capable of inducing granulomatous dermatitis and highlights the importance of systematically reviewing medication history when patients present with compatible cutaneous lesions.

Treatment of RGD depends on the underlying etiology. In cases associated with a systemic condition, control of the underlying disease can lead to improvement of PNGD and IGD, whereas in drug-induced forms, management often involves withdrawal or substitution of the offending agent. Additional treatments, such as topical or systemic corticosteroids, nonsteroidal anti-inflammatory drugs, hydroxychloroquine, colchicine, methotrexate, and JAK inhibitors may be necessary for persistent or severe cases. Although some patients with IGD experience spontaneous remission, others may develop recalcitrant disease; therefore, treatment must be tailored to disease severity and underlying comorbidities. Because RGD can be associated with systemic disorders, including hematologic and solid malignancies, rheumatologic diseases, and chronic infections, close clinical follow-up is recommended, even if initial evaluations are negative. Approximately 25% of patients may have no comorbidities at the time of diagnosis but later develop significant systemic conditions.^8^^,^^9^

This case adds dulaglutide to the list of medications associated with IGD. As dulaglutide use continues to expand in the management of type 2 diabetes, clinicians should remain vigilant for atypical cutaneous reactions. Additional case reports are warranted to establish the true incidence and risk factors for dulaglutide-induced IGD and to determine whether other GLP-1 receptor agonists (such as semaglutide and tirzepatide, increasingly used for weight-loss indications) pose a similar risk. In any patient presenting with clinical and histological findings suggestive of RGD, a thorough review of the medication history is essential. Early recognition of this potential association may prevent misdiagnosis, unnecessary treatments, and delays in discontinuing the offending agent.

### Declaration of generative AI and AI-assisted technologies in the writing process

None.

## Conflicts of interest

None disclosed.
